# Clinical Evaluation of Composite Restorations Using the Fédération Dentaire Internationale (FDI) Criteria: A Retrospective Cross-Sectional Study at University Dental Hospital, The University of Lahore, Pakistan

**DOI:** 10.7759/cureus.83582

**Published:** 2025-05-06

**Authors:** Fariha Naz, Malik Adeel Anwar, Muhammad Qasim, Shashit Shetty Bavabeedu, Shafait Ullah Khateeb, Ashraf Abdelfattah Khalil, Sadaf Inam, Syeda Sehar Nafees, Rehma Sammar

**Affiliations:** 1 Department of Operative and Pediatric Dentistry, University College of Medicine and Dentistry, The University of Lahore, Lahore, PAK; 2 Department of Oral Pathology and Oral Diagnostics, University College of Medicine and Dentistry, The University of Lahore, Lahore, PAK; 3 Department of Biomedical Engineering, Binghamton University (State University of New York), Binghamton, USA; 4 Department of Operative Dentistry and Endodontics, College of Dentistry, King Khalid University, Abha, SAU; 5 Department of Restorative Dental Sciences, College of Dentistry, King Khalid University, Abha, SAU

**Keywords:** composite resin, dental material, dental restoration failure, durability, esthetics

## Abstract

Background: Dental restorations play a very important role in maintaining oral function and aesthetics. However, the restoration’s longevity is influenced by various factors, including material properties, classification of cavities, and clinical performance over time. Understanding why the restorations fail is essential not only for better treatment results but also for improving their longevity. The Fédération Dentaire Internationale (FDI) criteria provide a detailed framework for assessing restoration performance in functional, biological, and aesthetic aspects.

Objective: This study aims to evaluate clinical success and failure rates using FDI criteria for dental restorations based on tooth location, cavity type, and restoration duration.

Methods: A retrospective cross-sectional clinical evaluation was conducted on 230 restored teeth in patients treated with permanent composite restoration. Restorations were grouped by location (anterior or posterior), duration (≤6 months, >6-12 months, >1-3 years, >3-5 years, >5 years), and cavity classification (Classes I-V). Functional (fracture of material and retention, marginal adaptation, proximal contact point), biological (caries at restoration margin, dental hard tissue defects, post-operative sensitivity), and aesthetic (surface luster and texture, color match, marginal staining) parameters were evaluated. A statistical analysis was conducted using Statistical Product and Service Solutions (SPSS, version 25; IBM SPSS Statistics for Windows, Armonk, NY) to determine significant differences.

Results: The overall success rate was 30% (n=69), while 70% (n=161) experienced failure. Success rates were comparable between anterior (29.2%, n=14) and posterior teeth (30.2%, n=55). Class I restorations had the highest success rate (42.4%, n=36), whereas Class V restorations showed the lowest (11.1%, n=1), with a statistically significant association between cavity classification and restoration longevity (p=0.012). Restoration success declined over time, with those exceeding five years showing the highest failure rate (81.5%, n=22). Functional evaluation showed a decline in fracture resistance and marginal adaptation over time, while biological assessment revealed an increase in secondary caries. Aesthetic factors, including color match and marginal staining, also worsened with time.

Conclusion: Restoration longevity is influenced by cavity classification and duration of the restoration, with Class I restorations exhibiting superior durability. The findings emphasize the progressive decline in functional, biological, and aesthetic properties over time, stressing the need for improved restorative materials and techniques to enhance long-term success.

## Introduction

Restorative dentistry has undergone significant advancements, improving both the patient outcomes and treatment longevity. Among different restorative options, composite restorations stand out due to their superior esthetic appeal and direct bonding ability [1]. However, the long-term durability of composite restorations is impacted by various factors, such as the clinician’s experience, material choice for filling, and correct clinical judgement. The interaction of these factors decides how long the restoration will last, making regular monitoring and maintenance an important part to ensure optimal clinical performance [2].

Composite material is considered a superior filling material with several advantages; however, restorations often fail due to factors such as secondary caries, fractures, marginal adaptation issues, and color mismatches [3]. A 2024 systematic review by Pousette Lundgren et al. [4] on composite restoration failures showed that the survival of these restorations depends on the patient’s age, material properties, and dentists' clinical experience. The study also reported lower success in patients with amelogenesis imperfecta, which means that a careful clinical assessment is required in these cases [4]. Similarly, patients with oro-facial clefts usually have dental anomalies that make restorative planning difficult and affect how long the restoration lasts [5].

Recent studies have calculated failure rates of composite restorations. Hofsteenge et al. reported an annual failure rate (AFR) between 1.5% and 4.9% over 4.6-22 years with restorations on multiple surfaces having more risk of failure [6]. Al-Asmar et al. found that 29.18% of the failed cases were from color mismatch and 9.8% were from poor anatomical shape [7]. Similarly, other studies show that restorations often need replacement due to various clinical problems that arise, such as marginal adaptation, secondary caries, and fractures [8,9].

The Fédération Dentaire Internationale (FDI) criteria are now a global standard to assess composite restorations. A 2023 review by Bresser et al. found that the most checked parameters for a composite restoration were marginal adaptation, staining, fractures, retention, recurrent caries, and postoperative sensitivity as the most common assessed parameters [10]. Additionally, restorations that followed FDI guidelines for anatomical form and color were linked to better patient satisfaction [11].

The evaluation methods for composite restorations have evolved a lot over the years. Earlier, assessments were based on the United States Public Health Service (USPHS) criteria established by Cvar et al. in 1971, which gave a structured but limited approach to evaluating restorations [12]. The FDI adopted a more structured approach for evaluating restorations by dividing the assessment into three parts: aesthetic, functional, and biological [13]. This detailed approach has become more common and was accepted by over half of the dental community by 2016 [6].

Newer developments in restorative dentistry have helped improve decision-making on whether to repair or replace a restoration. A 2024 study by Galina et al. reported that using FDI scores helped avoid unnecessary replacements, improving restoration longevity and lowering cost [14]. Similarly, a 2023 meta-analysis by Josic et al. confirmed that marginal integrity and discoloration are two key reasons why restoration fails [2].

The FDI criteria give a standardized framework to assess restorations, making an impact on treatment planning, repair, and patient satisfaction. An increasing body of literature supports their continued use to ensure consistency and reliability in clinical evaluations. More and more studies published serve as evidence that such criteria should be kept for use to guarantee uniformity and dependability in clinical assessment. This study aims to assess the clinical performance of composite restorations over an extended period and determine the failure rates using FDI criteria. Additionally, this study seeks to improve clinical decision-making practice regarding the necessity of restoration repair or replacement using FDI standards to ensure consistency in treatment planning and enhance overall patient care.

## Materials and methods

This retrospective cross-sectional clinical study was conducted in the Department of Operative Dentistry, University College of Dentistry, The University of Lahore. The study was approved by the Institutional Review Board of University College of Dentistry, The University of Lahore, under approval number UCD/ERCA/24/902. The duration of the study was three months, spanning from 1st October 2024 to 31st December 2024. Written informed consent was taken from all participants before their inclusion in the study.

The eligibility criteria included patients aged 15 years or older with at least one tooth restored with direct composite material and no history of dental examination within the past six months. A non-probability convenience sampling technique was used, where eligible patients who visited the dental hospital during the study period and met the inclusion criteria were consecutively recruited. A total of 230 restorations met the inclusion criteria and were analyzed. Each restoration was assessed based on nine parameters, divided into three domains:

*Functional parameters: *Fracture of material and retention, marginal adaptation, and proximal contact point

*Biological parameters: *Caries at restoration margin, dental hard tissue defects at restoration margin, and post-operative hypersensitivity/pulp status

*Aesthetic parameters:* Surface luster and texture, marginal staining, and color match

The evaluation system categorized restorations as follows: Clinically excellent (*FDI score 1*): No issues with function, biology, or aesthetics; Clinically good (*FDI score 2*): Minor deviations not requiring intervention; Clinically satisfactory (*FDI score 3*): Aesthetic or functional compromise; Clinically unsatisfactory (*FDI score 4*): Requiring immediate repair but not a full replacement; Clinically poor (*FDI score 5*): Needing a complete replacement; Not applicable (*FDI score 6*): Cases where a criterion could not be assessed were noted separately [15].

In this present study, the scoring system used was proposed by Hickel et al., where FDI scores 1-3 represented “Clinically acceptable” restorations, while FDI scores 4 and 5 denoted “Clinically unacceptable” cases and FDI score 6 as "Not applicable." To calculate the success rate, the data were dichotomized into sufficient (scores 1-3) and insufficient (scores 4 and 5) categories [15,16].

Restorations were labelled as successful if they received scores 1, 2, and 3 across all FDI criteria and failed if any criterion was scored 4 or 5 [15,16].

Key variables recorded for each restored tooth included its location (anterior or posterior), the duration since restoration placement (categorized as ≤6 months, >6-12 months, >1-3 years, >3-5 years, or >5 years), and the cavity classification based on GV Black’s system (cavity Class I, II, III, IV, or V). Clinical evaluations were conducted using both visual and tactile assessments.

The restorations evaluated in this study were originally placed by various dental practitioners at different clinics prior to the patients’ visits to the University Dental Hospital, The University of Lahore. Therefore, information regarding the specific clinician, the materials used, the bonding protocols, and isolation techniques during the original placement of restorations was not available.

For statistical analysis, qualitative variables were analyzed by frequencies and percentages. The chi-square test was used to study the association between qualitative variables. P-value <0.05 was considered statistically significant. All data analysis was performed using Statistical Product and Service Solutions (SPSS, version 25; IBM SPSS Statistics for Windows, Armonk, NY).

## Results

The study indicates a higher proportion of females as compared to males. Composite restorations were placed predominantly in posterior teeth. Class II restorations were the most common, followed by Class I, while Class III, IV, and V restorations were less frequent. Most restorations were placed within the last six months, with a smaller peak occurring between >1 and three years (Table 1).

**Table 1:** Demographic and clinical characteristics of the restored teeth

Category	N (%)
Gender	
Male	91 (39.6%)
Female	139 (60.4%)
Location of teeth	
Anterior	48 (20.9%)
Posterior	182 (79.1%)
Cavity class of restoration	
Class I	85 (37%)
Class II	102 (44.3%)
Class III	19 (8.3%)
Class IV	15 (6.5%)
Class V	9 (3.9%)
Duration of restoration	
≤6 Months	81 (35.2%)
>6-12 Months	26 (11.3%)
>1-3 Years	58 (25.3%)
>3-5 Years	38 (16.5%)
>5 Years	27 (11.7%)

Functional, biological, and aesthetic properties

Assessment of dental restorations based on functional, biological, and aesthetic qualities revealed that most criteria, including caries at restoration margin and dental hard tissue defects, with a high frequency falling in the “Clinically excellent” and “Clinically good” categories. However, marginal adaptation, material retention, and surface luster showed significant percentages in the “Clinically unsatisfactory” and “Clinically poor” categories. Proximal contact points had the highest “Not applicable” cases (Table 2).

**Table 2:** Assessment of composite restorations based on the Fédération Dentaire Internationale (FDI) criteria

	Assessment Parameter	Clinically Excellent (n, %)	Clinically Good (n, %)	Clinically Satisfactory (n, %)	Clinically Unsatisfactory (n, %)	Clinically Poor (n, %)	Not Applicable (n, %)
Functional	Fracture of material and retention	51 (22.20%)	33 (14.30%)	59 (25.70%)	59 (25.70%)	27 (11.70)	1 (0.40%)
	Marginal adaptation	27 (11.70%)	62 (27.00%)	51 (22.20%)	58 (25.20%)	30 (13.00%)	2 (0.90%)
	Proximal contact point	10 (4.30%)	28 (12.20%)	28 (13.00%)	32 (13.90%)	24 (10.40%)	106 (46.10%)
Biological	Caries at the restoration margin	79 (34.30%)	36 (15.70%)	57 (24.80%)	46 (20.00%)	12 (5.20%)	0 (0.00%)
	Dental hard tissue defects at the restoration margin	93 (40.40%)	48 (20.40%)	41 (17.80%)	34 (14.80%)	14 (6.10%)	0 (0.00%)
	Post-operative hypersensitivity/pulp status	48 (20.90%)	57 (24.80%)	38 (16.50%)	25 (10.90%)	13 (5.70%)	49 (21.30%)
Aesthetic	Surface luster & texture	35 (15.20%)	59 (25.70%)	56 (24.30%)	49 (21.30%)	16 (7.00%)	15 (6.50%)
	Marginal staining	60 (26.10%)	36 (15.70%)	54 (23.50%)	48 (20.90%)	17 (7.40%)	15 (6.50%)
	Color match	23 (10.00%)	63 (27.40%)	68 (29.60%)	39 (17.00%)	21 (9.10%)	16 (7.00%)

Functional, biological, and aesthetic properties with restoration duration

Table 3 presents a comparative analysis of the functional, biological, and aesthetic characteristics of dental restorations over time. A progressive decline in material fracture resistance and retention was observed, with the proportion of clinically acceptable restorations decreasing from 70.4% (n=57) in those under six months old to 51.9% (n=14) in those exceeding five years. Similarly, marginal adaptation exhibited a significant decline, reducing from 70.4% (n=57) to 48.1% (n=13) over the same period.

**Table 3:** Comparison of functional, biological, and aesthetic properties in relation to restoration duration

	Assessment Parameter	Evaluation Criteria	≤6 Months	>6-12 Months	>1-3 Years	>3-5 Years	>5 Years	Chi-Square Test	p-value
Functional	Fracture of material and retention	Clinically Acceptable	57 (70.40%)	14 (53.80%)	37 (63.80%)	21 (55.30%)	14 (51.90%)	12.231	0.141
		Clinically Unacceptable	24 (29.60%)	12 (46.20%)	21 (36.20%)	17 (44.70%)	12 (44.40%)		
		Not Applicable	0 (0.00%)	0 (0.00%)	0 (0.00%)	0 (0.00%)	1 (3.70%)		
	Marginal adaptation	Clinically Acceptable	57 (70.40%)	14 (53.80%)	35 (60.30%)	21 (55.30%)	13 (48.10%)	8.738	0.365
		Clinically Unacceptable	23 (28.40%)	12 (46.20%)	23 (39.70%)	16 (42.10%)	14 (51.90%)		
		Not Applicable	1 (1.20%)	0 (0.00%)	0 (0.00%)	1 (2.60%)	0 (0.00%)		
	Proximal contact point	Clinically Acceptable	29 (35.8%)	8 (30.8%)	14 (24.1%)	10 (26.3%)	7 (25.9%)	13.509	0.095
		Clinically Unacceptable	14 (17.3%)	10 (38.50%)	15 (25.9%)	6 (15.80%)	11 (40.7%)		
		Not Applicable	38 (46.9%)	8 (30.8%)	29 (50.0%)	22 (57.90%)	9 (33.3%)		
Biological	Caries at restoration margin	Clinically Acceptable	69 (85.20%)	23 (88.50%)	36 (62.10%)	28 (73.70%)	16 (59.30%)	15.673	0.003
		Clinically Unacceptable	12 (14.80%)	3 (11.50%)	22 (37.90%)	10 (26.30%)	11 (40.70%)		
		Not Applicable	0 (0.00%)	0 (0.00%)	0 (0.00%)	0 (0.00%)	0 (0.00%)		
	Dental hard tissue defects at restoration margin	Clinically Acceptable	73 (90.10%)	19 (73.10%)	42 (72.40%)	30 (78.90%)	18 (66.70%)	10.629	0.031
		Clinically Unacceptable	8 (9.90%)	7 (26.90%)	16 (27.60%)	8 (21.10%)	9 (33.30%)		
		Not Applicable	0 (0.00%)	0 (0.00%)	0 (0.00%)	0 (0.00%)	0 (0.00%)		
	Post-operative hypersensitivity/Pulp Status	Clinically Acceptable	54 (66.70%)	14 (53.80%)	35 (60.20%)	24 (63.20%)	16 (59.30%)	7.346	0.500
		Clinically Unacceptable	9 (11.10%)	6 (23.10%)	10 (17.20%)	5 (13.20%)	8 (29.60%)		
		Not Applicable	18 (22.20%)	6 (23.10%)	13 (22.40%)	9 (23.70%)	3 (11.10%)		
Aesthetic	Surface luster and texture	Clinically Acceptable	59 (72.80%)	16 (61.50%)	34 (58.6%)	26 (68.4%)	15 (55.6%)	6.263	0.618
		Clinically Unacceptable	16 (19.80%)	8 (30.80%)	21 (36.2%)	10 (26.3%)	10 (37.0%)		
		Not Applicable	6 (7.40%)	2 (7.70%)	3 (5.2%)	2 (5.3%)	2 (7.4%)		
	Marginal staining	Clinically Acceptable	64 (79.0%)	15 (57.7%)	33 (56.9%)	22 (57.9%)	16 (59.3%)	13.807	0.087
		Clinically Unacceptable	11 (13.6%)	9 (34.6%)	22 (37.9%)	14 (36.8%)	9 (33.3%)		
		Not Applicable	6 (7.4%)	2 (7.7%)	3 (5.2%)	2 (5.3%)	2 (7.4%)		
	Color match	Clinically Acceptable	62 (76.5%)	15 (57.7%)	36 (62.1%)	27 (71.1%)	14 (51.9%)	8.823	0.357
		Clinically Unacceptable	14 (17.3%)	9 (34.6%)	17 (29.3%)	9 (23.7%)	11 (40.7%)		
		Not Applicable	5 (6.2%)	2 (7.7%)	5 (8.6%)	2 (5.3%)	2 (7.4%)		

Biological properties also showed notable deterioration. Caries at the margin of the restoration had a statistically significant deteriorating trend (p=0.003), indicating increased susceptibility to aging. Additionally, dental hard tissue defects significantly increased over time (p=0.031), suggesting progressive structural deterioration.

In contrast, aesthetic parameters, including surface luster, marginal discoloration, and color, declined with age but were statistically insignificant (p>0.05), indicating more subjective or gradual changes.

Overall, the findings suggest that, while restoration maintains a degree of functionality over time, its durability and biological integrity diminish, particularly in fracture resistance, marginal fitness, and cariogenic susceptibility.

Functional, biological, and aesthetic properties of teeth location

Table 4 summarizes a comparative analysis of the functional, biological, and aesthetic characteristics of anterior and posterior dental restorations. Material fracture resistance and retention were slightly higher in anterior restorations (66.7%, n=32) than in posterior restorations (61.0%, n=111), though the difference was statistically insignificant (p=0.695). Similarly, marginal adaptation and proximal contact points showed minor improvements in anterior restorations but without significant variation.

**Table 4:** Comparison of functional, biological, and aesthetic properties between anterior and posterior restorations

	Assessment Parameter	Evaluation Criteria	Anterior (n, %)	Posterior (n, %)	Chi square test	P-value
Functional	Fracture of material and retention	Clinically Acceptable	32 (66.70%)	111 (61.00%)	0.728	0.695
		Clinically Unacceptable	16 (33.30%)	70 (38.50%)		
		Not Applicable	0 (0.00%)	1 (0.50%)		
	Marginal adaptation	Clinically Acceptable	31 (64.60%)	109 (59.90%)	0.793	0.673
		Clinically Unacceptable	17 (35.40%)	71 (39.00%)		
		Not Applicable	0 (0.00%)	2 (1.10%)		
	Proximal contact point	Clinically Acceptable	19 (39.60%)	49 (26.90%)	3.097	0.213
		Clinically Unacceptable	9 (18.80%)	47 (25.80%)		
		Not Applicable	20 (41.60%)	86 (47.30%)		
Biological	Caries at the restoration margin	Clinically Acceptable	38 (79.2%)	134 (73.6%)	0.618	0.432
		Clinically Unacceptable	10 (20.8%)	48 (26.4%)		
		Not Applicable	0 (0.00%)	0 (0.00%)		
	Dental hard tissue defects at the restoration margin	Clinically Acceptable	37 (77.1%)	145 (79.7%)	0.154	0.695
		Clinically Unacceptable	11 (22.9%)	37 (20.3%)		
		Not Applicable	0 (0.00%)	0 (0.00%)		
	Post-operative hypersensitivity/pulp status	Clinically Acceptable	28 (58.3%)	115 (63.2%)	5.526	0.063
		Clinically Unacceptable	13 (27.1%)	25 (13.7%)		
		Not Applicable	7 (14.6%)	42 (23.1%)		
Aesthetic	Surface luster and texture	Clinically Acceptable	32 (66.7%)	118 (64.8%)	0.056	0.972
		Clinically Unacceptable	13 (27.1%)	52 (28.6%)		
		Not Applicable	3 (6.3%)	12 (6.6%)		
	Marginal staining	Clinically Acceptable	30 (62.5%)	120 (65.9%)	0.267	0.875
		Clinically Unacceptable	15 (31.3%)	50 (27.5%)		
		Not Applicable	3 (6.3%)	12 (6.6%)		
	Color match	Clinically Acceptable	34 (70.8%)	120 (65.9%)	0.413	0.813
		Clinically Unacceptable	11 (22.9%)	49 (26.9%)		
		Not Applicable	3 (6.3%)	13 (7.1%)		

Biological factors revealed a high incidence of caries at the restoration margin in posterior teeth (73.6%, n=134) compared to anterior teeth (79.2%, n=38). Postoperative hypersensitivity was frequently reported in posterior restorations 63.2%, n=115) compared to anterior restorations (58.3%, n=28).

Regarding aesthetic properties, such as surface luster, marginal discoloration, and color compatibility, no significant difference was observed in anterior and posterior restorations, suggesting comparable outcomes in both regions.

Overall, while minor differences exist, functional and aesthetic characteristics remain similar between anterior and posterior restorations with no statistically significant variations.

Functional, biological, and aesthetic properties of cavity class

Table 5 summarizes the functional, biological, and aesthetic properties of dental restorations by cavity class. Class I restorations exhibit the highest clinical acceptability, particularly in fracture resistance (81.2%, n=69), marginal adaptation (83.5%, n=71), and Class V restorations show the poorest performance with high rates of clinical unacceptability in these aspects.

**Table 5:** Comparison of functional, biological, and aesthetic properties across cavity classes

	Assessment Parameter	Evaluation Criteria	Class-I (n, %)	Class-II (n, %)	Class-III (n, %)	Class-IV (n, %)	Class-V (n, %)	Chi-square test	P-value
Functional	Fracture of material and retention	Clinically Acceptable	69 (81.2%)	46 (45.1%)	13 (68.4%)	13 (86.7%)	2 (22.2%)	38.949	<0.001
		Clinically Unacceptable	15 (17.6%)	56 (54.9%)	6 (31.6%)	2 (13.3%)	7 (77.8%)		
		Not Applicable	1 (1.2%)	0 (0.0%)	0 (0.0%)	0 (0.0%)	0 (0.0%)		
	Marginal adaptation	Clinically Acceptable	71 (83.5%)	44 (43.1%)	10 (52.6%)	13 (86.7%)	2 (22.2%)	49.031	<0.001
		Clinically Unacceptable	13 (15.3%)	58 (56.9%)	8 (42.1%)	2 (13.3%)	7 (77.8%)		
		Not Applicable	1 (1.2%)	0 (0.0%)	1 (5.3%)	0 (0.0%)	0 (0.0%)		
	Proximal contact point	Clinically Acceptable	0 (0.0%)	48 (47.1%)	13 (68.4%)	7 (46.7%)	0 (0.0%)	190.689	<0.001
		Clinically Unacceptable	0 (0.0%)	45 (44.1%)	6 (31.6%)	5 (33.3%)	0 (0.0%)		
		Not Applicable	85 (100.0%)	9 (8.8%)	0 (0.0%)	3 (20.0%)	9 (100.0%)		
Biological	Caries at the restoration margin	Clinically Acceptable	73 (85.9%)	68 (66.7%)	14 (73.7%)	12 (80.0%)	5 (55.6%)	11.109	0.025
		Clinically Unacceptable	12 (14.1%)	34 (33.3%)	5 (26.3%)	3 (20.0%)	4 (44.4%)		
		Not Applicable	0 (0.0%)	0 (0.0%)	0 (0.0%)	0 (0.0%)	0 (0.0%)		
	Dental hard tissue defects at the restoration margin	Clinically Acceptable	77 (90.6%)	74 (72.5%)	15 (78.9%)	12 (80.0%)	4 (44.4%)	15.997	0.003
		Clinically Unacceptable	8 (9.4%)	28 (27.5%)	4 (21.1%)	3 (20.0%)	5 (55.6%)		
		Not Applicable	0 (0.0%)	0 (0.0%)	0 (0.0%)	0 (0.0%)	0 (0.0%)		
	Post-operative hypersensitivity/pulp status	Clinically Acceptable	64 (75.3%)	50 (49.0%)	11 (57.9%)	13 (86.7%)	5 (55.6%)	33.405	<0.001
		Clinically Unacceptable	8 (9.4%)	18 (17.6%)	7 (36.8%)	1 (6.7%)	4 (44.4%)		
		Not Applicable	13 (15.3%)	34 (33.3%)	1 (5.3%)	1 (6.7%)	0 (0.0%)		
Aesthetic	Surface luster and texture	Clinically Acceptable	71 (83.5%)	51 (50.0%)	16 (84.2%)	10 (66.7%)	2 (22.2%)	35.756	<0.001
		Clinically Unacceptable	12 (14.1%)	41 (40.2%)	2 (10.5%)	5 (33.3%)	5 (55.6%)		
		Not Applicable	2 (2.4%)	10 (9.8%)	1 (5.3%)	0 (0.0%)	2 (22.2%)		
	Marginal staining	Clinically Acceptable	64 (75.3%)	61 (59.8%)	15 (78.9%)	9 (60.0%)	1 (11.1%)	22.498	0.004
		Clinically Unacceptable	19 (22.4%)	31 (30.4%)	3 (15.8%)	6 (40.0%)	6 (66.7%)		
		Not Applicable	2 (2.4%)	10 (9.8%)	1 (5.3%)	0 (0.0%)	2 (22.2%)		
	Color match	Clinically Acceptable	68 (80.0%)	56 (54.9%)	13 (68.4%)	13 (86.7%)	4 (44.4%)	20.136	0.010
		Clinically Unacceptable	14 (16.5%)	36 (35.3%)	5 (26.3%)	2 (13.3%)	3 (33.3%)		
		Not Applicable	3 (3.5%)	10 (9.8%)	1 (5.3%)	0 (0.0%)	2 (22.2%)		

Statistically significant differences (p<0.001) were observed in fracture resistance, marginal adaptation, and post-operative hypersensitivity, indicating that cavity class significantly influences these properties. While Class III and IV restorations show moderate success, they have higher rates of unacceptable marginal adaptation and proximal contact point deficiencies.

Caries formation at the marginal to the restoration is notably more prevalent in Class V restorations (p=0.025), while the extent of dental hard tissue loss increases with cavity complexity (p=0.003). Aesthetic factors, such as marginal color match and marginal staining, are generally less well tolerated with Class I restorations but deteriorate as cavity complexity increases (p=0.004 and p=0.010, respectively). These findings highlight that the long-term success of restorations is significantly influenced by cavity classification.

The overall success rate of restoration is 30% (n=69), with a failure rate of 70% (n=161). Success rates were comparable between anterior and posterior teeth, showing no statistically significant difference. Class I restorations had the highest success, while Class V had the lowest. A statistically significant difference (p=0.012) suggests that cavity class impacts restoration longevity. The success rate declined over time, with restorations older than five years failing the most. While this trend was notable, statistical significance was not reached (0.057) (Table 6).

**Table 6:** Association of restoration with tooth location, cavity, class, and duration

	Success n(%)	Failure n(%)	Chi-square test	P-value
Location of teeth				
Anterior	14 (29.20%)	34 (70.80%)	0.020	0.887
Posterior	55 (30.20%)	127 (69.80%)		
Cavity class of restoration				
Class I	36 (42.40%)	49 (57.60%)	12.845	0.012
Class II	21 (20.60%)	81 (79.40%)		
Class III	5 (26.30%)	14 (73.70%)		
Class IV	6 (40%)	9 (60%)		
Class V	1 (11.10%)	8 (88.90%)		
Duration of restoration				
≤ 6 Months	34 (42%)	47 (58%)	9.179	0.057
>6-12 Months	7 (26.90%)	19 (73.10%)		
>1-3 Years	13 (22.40%)	45 (77.60%)		
>3-5 Years	10 (26.30%)	28 (73.70%)		
> 5 Years	5 (18.50%)	22 (81.50%)		
Overall Rate	69 (30%)	161 (70%)	-	-

## Discussion

The selection of FDI criteria in this study is based on established literature, which indicates that researchers commonly adapt and selectively use these criteria to study objectives. Prior studies have shown that, on average, only 8.5 out of the 16 FDI criteria are utilized, with a strong emphasis on functional, biological, and aesthetic aspects. Specifically, criteria such as marginal adaptation, fracture of material, retention, and recurrence of caries are among the most frequently applied as they provide reliable and clinically relevant assessments. Additionally, some criteria, including radiographic examination and patient-reported outcomes, have been deemed either irrelevant or difficult to assess consistently [13,17,18].

A significant finding of the study was that lack of marginal adaptation issues were the most common cause of restoration failure, with 25.2% of restorations deemed clinically unsatisfactory and 13% classified as poor. This finding aligns with Josic et al., who identified marginal integrity and discoloration as major predictors of restoration failure [2]. However, Bresser et al. registered a considerably lower marginal adaptation failure of 10%, suggesting that differences in bonding protocols, operator skill, or patient-specific factors could contribute to variability in failure rates [10]. Factors such as improper bonding procedures and polymerization shrinkage may have played a significant role in the high marginal adaptation failure rate observed in this study.

Early failures were a notable concern, with 58% of the restorations failing within the first six months. This aligns with existing literature, which suggests that procedural mistakes, weak bonding, and polymerization shrinkage are major reasons for early failure. Comparatively, Hofsteenge et al. reported an AFR between 1.5% and 4.9% over a period of 4.6-22 years. Their study found that restorations involving two or more surfaces were much more prone to failure [6]. Similarly, Opdam et al. reported that posterior composite restorations had a 10-year median survival with AFRs ranging from 1% to 3% [3]. These differences point out the need to refine bonding protocols and improve moisture control strategies to increase restoration longevity.

When it comes to how long the restoration lasts, failure rates increased over time, with 81% of restorations failing after five years. This pattern indicates the combined effects of recurrent caries, marginal breakdown, and wear-related issues over time. Similarly, recurrent carriers are the main reason for restoration failure, with rates ranging from 5% to 30% depending on the age of restoration [13]. In the present study, the percentage of restorations free from marginal caries dropped from 85.2% in restorations that were placed within a period of less than six months to 59.3% in restorations older than five years. These results highlight the importance of regular monitoring and early interventions to prevent long-term failure.

The study found no statistically significant difference between failure rates of anterior (70%) and posterior (69.8%) composite restorations. This contradicts the findings of Hofsteenge et al., who reported higher failure rates in posterior teeth due to greater occlusal forces [6]. The difference suggests that factors such as operator technique, occlusal adjustments, and patient-specific variations in occlusal load may influence failure patterns. Future research should investigate how these variables affect restoration longevity.

In terms of cavity classification, Class V restorations exhibited the highest failure rates, followed by Class II restorations. The high failure rate of Class V restorations can be attributed to their location at the gingival margin, where moisture contamination and limited bonding surface pose challenges. This finding is consistent with Pousette Lundgren et al., who stressed the challenge of achieving durable adhesion in moist environments [4]. Conversely, Class I restorations demonstrated the lowest failure rates, a finding also supported by Opdam et al., whose meta-analysis concluded that simpler cavity forms lead to increased restoration longevity [3].

Aesthetic factors, such as surface luster, marginal color change, and color matching, deteriorated over time, but these differences were not statistically significant (p>0.05). Maillet et al. reported that 38.3% of restorations failed primarily due to color mismatch and anatomical discrepancies, while da Costa et al. [17] found that restorations meeting FDI specifications for anatomical and color attributes correlated with higher patient satisfaction [19]. While aesthetics was not a primary cause of failure in this study, maintaining long-term color stability remains an important consideration for clinical success.

The present study stresses the need for improved clinical protocols to enhance the longevity of composite restorations. Operator training, better bonding techniques, and enhanced moisture control could reduce early failures. As Galina et al. [14] suggested, intervention strategies based on FDI scores may help reduce unnecessary replacements, ensuring cost-effectiveness and patient satisfaction [10]. Additionally, future research should focus on large-scale long-term studies with controlled variables such as patient diet, occlusal forces, and material properties. Standardized failure definitions across studies would facilitate better comparability and improved clinical decision-making.

The present study evaluated the clinical performance of composite restorations based on the FDI criteria, finding 70% of restorations to be clinically unacceptable. This is notably higher than other studies, such as the study in France, which reported a 38.8% failure rate, and from Sudan, which found a 48% failure rate [19,20]. One reason for this discrepancy could be differences in failure definitions; whereas some studies only classify restorations requiring replacement as failures, the present study considered any restoration requiring immediate repair (but not full replacement) and those needing a complete replacement (FDI scores 4 and 5) as failures. Moreover, the higher failure rate observed may be influenced by differences in operator skill, bonding techniques, and environmental factors such as moisture contamination. Addressing these factors through improved clinical protocols and material selection could enhance restoration longevity and clinical outcomes.

This study has limitations, including its single-center setting, which may limit generalization. The cross-sectional design provides a snapshot of restoration performance but does not track changes over time. Additionally, since the restorations assessed were placed by various dental practitioners outside our institution, detailed information about the original operator skill, material used, and isolation techniques was not available, potentially introducing variability in the results. Uncontrolled variations in operator skill, patient compliance, and material selection may have influenced failure rates. Future research should focus on standardized assessment methods and well-controlled trials to gain a deeper understanding of composite restoration longevity.

## Conclusions

This study found that the majority of composite restorations were clinically unacceptable based on the FDI criteria, with marginal adaptation being the most common reason for failure. Early failures were mostly concerning, as more than half of the restorations failed within the first six months, showing the strong influence of bonding techniques and procedural factors. Over time, failure rates increased, with most restorations failing after five years, mainly due to recurrent caries and marginal deterioration.

No significant difference was found between anterior and posterior restorations, suggesting that failure is not solely linked to occlusal load. Class V restorations had the highest failure rates, most likely due to challenges caused by bonding at the gingival margin. Although aesthetic factors declined over time, they were not the primary cause of restoration failure. These results reinforce the need for better clinical protocols, improved moisture control, and enhanced operator training to extend the longevity of composite restorations.

## References

[1] Tiron B, Forna N, Topoliceanu C (2023). Assessment of factors influencing the esthetic, functional and biological status of posterior composite resins restorations. Rom J Oral Rehabil.

[2] Josic U, D'Alessandro C, Miletic V (2023). Clinical longevity of direct and indirect posterior resin composite restorations: an updated systematic review and meta-analysis. Dent Mater.

[3] Opdam NJ, van de Sande FH, Bronkhorst E (2014). Longevity of posterior composite restorations: a systematic review and meta-analysis. J Dent Res.

[4] Pousette Lundgren G, Dahllöf G (2024). Advances in clinical diagnosis and management of amelogenesis imperfecta in children and adolescents. J Dent.

[5] Huda NU, Shahzad HB, Noor M, Ishaq Y, Anwar MA, Kashif M (2021). Frequency of different dental irregularities associated with cleft lip and palate in a tertiary care dental hospital. Cureus.

[6] Hofsteenge JW, Scholtanus JD, Özcan M, Nolte IM, Cune MS, Gresnigt MM (2023). Clinical longevity of extensive direct resin composite restorations after amalgam replacement with a mean follow-up of 15 years. J Dent.

[7] Al-Asmar AA, Sabrah AH, Ismail NH, Abd-Raheam IM, Oweis YG (2023). Clinical evaluation of reasons for immediate composite restoration failure placed by dental students: a cross-sectional study in Jordan. Open Dent J.

[8] Alhoshani RN, Khan AM (2024). Assessment of prevalence and causative factors for replacement of composite and amalgam restorations: an observational study. J Pharm Bioallied Sci.

[9] Costa MB, Tomisaki ET, Mendonça dos Santos DC, Hoeppner MG, Cardoso SA (2021). Clinical evaluation of composite resin restorations in posterior teeth. J Health Sci.

[10] Bresser RA, Hofsteenge JW, Wieringa TH, Braun PG, Cune MS, Özcan M, Gresnigt MM (2023). Clinical longevity of intracoronal restorations made of gold, lithium disilicate, leucite, and indirect resin composite: a systematic review and meta-analysis. Clin Oral Investig.

[11] Freitas BN, Silva POD, Pintado-Palomino K (2023). Patients´ satisfaction concerning direct anterior dental restoration. Braz Dent J.

[12] Cvar JF, Ryge G (2005). Reprint of criteria for the clinical evaluation of dental restorative materials. 1971. Clin Oral Investig.

[13] Marquillier T, Doméjean S, Le Clerc J (2018). The use of FDI criteria in clinical trials on direct dental restorations: a scoping review. J Dent.

[14] Galina P, Andrei G, Antonia M (2024). Non-intervention versus repair/replacement decisions in posterior composite restorations aged 3-5 years: a retrospective study. Rom J Oral Rehabil.

[15] Hickel R, Peschke A, Tyas M (2010). FDI World Dental Federation - clinical criteria for the evaluation of direct and indirect restorations. Update and clinical examples. J Adhes Dent.

[16] Hickel R, Roulet JF, Bayne S (2007). Recommendations for conducting controlled clinical studies of dental restorative materials. Clin Oral Investig.

[17] da Costa TR, Ferri LD, Loguercio AD, Reis A (2014). Eighteen-month randomized clinical trial on the performance of two etch-and-rinse adhesives in non-carious cervical lesions. Am J Dent.

[18] Paula EA, Tay LY, Kose C, Mena-Serrano A, Reis A, Perdigão J, Loguercio AD (2015). Randomized clinical trial of four adhesion strategies in cervical lesions: 12-month results. Int J Esthet Dent.

[19] Maillet C, Decup F, Dantony E (2022). Selected and simplified FDI criteria for assessment of restorations. J Dent.

[20] Ijaimi ZA, Abu-Bakr NH, Ibrahim YE (2015). Assessment of the quality of composite resin restorations. OJST.

